# Family history of cancer is a prognostic factor for better survival in operable esophageal squamous cell carcinoma: A propensity score matching analysis

**DOI:** 10.3389/fonc.2022.945937

**Published:** 2022-12-14

**Authors:** Shuishen Zhang, Junying Chen, Bin Li, Xiaoli Cai, Kexi Wang, Zihui Tan, Yuzhen Zheng, Qianwen Liu

**Affiliations:** ^1^ Department of Thoracic Surgery, The First Affiliated Hospital, Sun Yat-Sen University, Guangzhou, China; ^2^ Guangdong Esophageal Cancer Institute, Guangzhou, China; ^3^ Department of Thoracic Oncology, Sun Yat-Sen University Cancer Center, Guangzhou, China; ^4^ State Key Laboratory of Oncology in South China, Collaborative Innovation Center for Cancer Medicine, Sun Yat-sen University Cancer Center, Guangzhou, China; ^5^ Biostatistics Team, Clinical Trials Unit, The First Affiliated Hospital, Sun Yat-sen University, Guangzhou, China; ^6^ Department of Medical Ultrasonics, First Affiliated Hospital of Jinan University, Guangzhou, China; ^7^ Department of Thoracic Surgery, Sun Yat-sen Memorial Hospital, Sun Yat-sen University, Guangzhou, China; ^8^ Department of Thoracic Surgery, The Six Affiliated Hospital, Sun Yat-sen University, Guangzhou, China

**Keywords:** esophageal squamous cell carcinoma, family history of cancer, digestive tract cancer, family history of cancer in first degree, prognosis

## Abstract

**Lay summary:**

Patients with a family history of cancer, especially digestive tract cancer and esophageal cancer, a family history of cancer in the first degree, and more than one relative affected by cancer were associated with favorable survival when compared to those without a family history of cancer.

**Precis for use in the Table of Contents:**

A family history of cancer is a favorable independent prognostic factor in ESCC. Patients with a family history of cancer, especially digestive tract cancer and esophageal cancer, a family history of cancer in the first degree, and more than one relative affected by cancer were associated with favorable survival when compared to those without a family history of cancer.

**Background:**

A family history of cancer (FH) is closely associated with the risk and survival of many cancers. However, the effect of FH on the prognosis of patients with esophageal squamous cell carcinoma (ESCC) remains unclear. We performed a large cohort study in the Chinese population to obtain insight into the prognostic value of FH in patients with operable ESCC.

**Methods:**

A total of 1,322 consecutive patients with thoracic ESCC who had undergone esophagectomy between January 1997 and December 2013 were included. The FH group included patients with any degree of FH, while the non-FH group included patients without any degree of FH. In total, 215 patients with FH and 215 without FH were matched using the propensity score matching analysis method to adjust for differences in baseline variables between the two groups. The impact of FH on disease-free survival (DFS) and overall survival (OS) was estimated using the Kaplan–Meier method and Cox’s proportional hazards models.

**Results:**

Before matching, 280 (21.2%) patients were included in the FH group and 1,042 (78.8%) in the non-FH group. FH was associated with early pathological T stage (*p* = 0.001), lymph node-negative status (*p* = 0.022), and early pathological stage (*p* = 0.006). After matching, FH was an independent prognostic factor for DFS and OS in ESCC patients. Patients with FH had 35% lower risk of disease progression (hazard ratio [HR] = 0.65, 95% CI: 0.51–0.84, *p* = 0.001) and 34% lower risk of death (HR = 0.66, 95% CI: 0.51–0.86, *p* = 0.002) than those without FH. Patients with a family history of digestive tract cancer (FH-DC), a family history of esophageal cancer (FH-EC), FH in first-degree relatives (FH-FD), and more than one relative affected by cancer were associated with favorable DFS and OS as compared to those without FH.

**Conclusion:**

FH is a favorable independent prognostic factor in ESCC. Patients with FH, especially those with FH-DC, FH-EC, FH-FD, and more than one relative affected by cancer, had improved survival.

## Introduction

Esophageal squamous cell carcinoma (ESCC), which is the predominant histopathology of esophageal cancer (EC), is one of the most common and aggressive cancers in China, with more than 252,000 new cases and 193,000 deaths annually (1). Despite decades of improvements in surgical techniques and the use of multiple therapeutic approaches, the survival of patients with ESCC remains unsatisfactory with a high rate of recurrence (2, 3). Accurately identifying ESCC patients who will experience recurrence or progression remains difficult. Therefore, identifying novel prognostic factors is quite important to determine patients who are at high risk.

Numerous studies have shown that a family history of cancer (FH) is closely associated with the risk and survival of many cancers, including breast (4), colorectal (5), and gastric cancers (6), as well as nasopharyngeal carcinoma (7). The first-degree family history of colorectal and gastric cancers as well as nasopharyngeal carcinoma was associated with improved survival (5–7). Moreover, breast cancer patients with a family history of breast cancer had a favorable prognosis. Many studies have provided convincing evidence that a family history of EC is a risk factor for the development of this disease (8–11). Therefore, this evidence raises the question of whether a family history of EC is a prognostic factor for patients with ESCC. Previously, only three studies explored the association between FH and the prognosis of ESCC and reported conflicting results. One study from Northern China found no significant difference in survival rates between patients with a family history of upper gastrointestinal cancer and sporadic cases after surgery. Familial cases had a significantly lower survival rate than sporadic cases for patients above the age of 50 years (12). However, a study from Western China reported that a family history of EC is an unfavorable prognostic factor in ESCC patients who had undergone surgery (13). Another study with a limited population from Southern China showed that ESCC patients with a first-degree FH, especially digestive tract cancer, had poor survival rates after radiotherapy (14). The conflicting results may be attributed to variations in strategies in selecting the study population, definitions of family history, or sample sizes. Assessing the prognostic value of FH in ESCC not only has potential value in outcome prediction but also is valuable in understanding the mechanisms of disease etiology and progression. Thus far, no study with a large population of ESCC patients has been performed that systematically elucidated the prognostic value of FH in ESCC patients according to cancer type, first-degree family history, and the number of affected relative members.

Information on the effect of FH, including a family history of EC, a family history of digestive tract cancer, FH in the first degree, and the number of affected relative members, on the prognosis of patients with operable ESCC is scarce and unclear. Therefore, we performed a large cohort study in the Chinese population to obtain insights into the prognostic value of FH according to cancer type, first-degree relatives, and the number of affected relatives in patients with operable ESCC.

## Materials and methods

### Patient selection

We identified consecutive patients with EC at Sun Yat-sen University Cancer Center between January 1997 and December 2013. Patients were included based on the following criteria: 1) newly confirmed to have thoracic ESCC and had not received any treatment prior to admission; had undergone right transthoracic esophagectomy; had undergone complete pretreatment evaluation including patient history, physical examination, and computed tomography of the neck, chest, and upper abdomen; had undergone endoscopic ultrasound; and had a complete interview about family history. Patients were excluded based on the following criteria: history of other cancer, prior neoadjuvant therapy, death in the perioperative period, and lack of information on FH. Pathologic stage was retrospectively determined according to the 7th edition of the American Joint Committee on Cancer staging system. All patients provided written informed consent for their information to be stored and used in the hospital database. Study approval was obtained from independent ethics committees at Sun Yat-Sen University Cancer Center.

### Clinicopathological factors

Clinicopathological factors associated with survival were collected from the patients’ medical records. These factors included age at primary diagnosis, gender, smoking, alcohol consumption, dysphagia, radicality of surgery, adjuvant therapy, pathological differentiation, tumor location, pathological (p) T stage, pN stage, and FH.

The definitions of smoking, alcohol consumption, and adjuvant therapy have been described in our previous study (15). Patients who had smoked more than 100 cigarettes in their lifetime were defined as smokers; those who smoked and stopped smoking more than 1 year before the time of admission to the hospital were defined as former smokers. Patients were routinely requested to report their lifetime history of drinking, including status, frequency, average consumption amount, and type of alcohol, at the time of admission. Former drinkers were defined as those who drank alcohol and discontinued drinking alcohol more than 1 year before the time of admission to the hospital; current drinkers were defined as those who still drank alcohol at the time of admission to the hospital or had stopped drinking alcohol within 1 year before the time of admission in the hospital. Adjuvant therapy is usually recommended for patients with lymph node (LN) metastasis. Treatment options were selected based on tumor stage, doctor’s opinion, patient’s performance status, and patient’s desire. Generally, adjuvant therapy was started 4–8 weeks after the operation. In this study, 29 patients received postoperative chemoradiotherapy, 54 patients received postoperative radiotherapy, and 204 patients received adjuvant chemotherapy.

### Family history assessment

FH was ascertained by interviewing patients themselves and/or their family members at the time of case diagnosis. A positive family history was classified according to cancer in first-degree or second-degree relatives and cancer type (esophageal and all other cancers). The FH group was defined as those patients who had any degree of FH. The non-FH group was defined as patients who did not have any degree of FH. A family history of esophageal cancer (FH-EC) was defined as any degree of a family history of EC. A family history of non-EC (FH-NEC) was defined as any degree of a family history of other cancer but not EC. A family history of digestive tract cancer (FH-DC) was defined as any degree of FH including esophageal, stomach, liver, pancreas, or colorectal cancers, while a family history of non-digestive tract cancer (FH-NDC) was defined as any degree of a family history of other cancer but not digestive tract cancer (11). FH in the first degree (FH-FD) was defined as parents, siblings, or offspring or first-degree relatives who had a family history of any cancer; FH in non-first-degree (FH-NFD) meant second- or third-degree relatives (aunt, uncle, niece, nephew, or grandparent) who had FH but not first-degree. If patients had a family history in several degrees of relatives, they were regarded as having FH in the closer degree in blood. Moreover, we recorded the number of any degree of family members with cancer.

### Follow-up and outcomes

All patients received standardized follow-ups at 3-month intervals for the first 2 years after surgery, a 6-month interval in the third year, and yearly thereafter. Follow-up time was calculated from the date of surgery to the event or the date of the last contact (16). Follow-up continued until June 2019. The outcomes in this study were overall survival (OS) and disease-free survival (DFS). OS was defined as the time from the date of surgery to all-cause death or the last contact. DFS was defined as the time from the date of surgery to the first recurrence of index cancer or all-cause death or the last contact, whichever occurred first.

### Propensity score matching

Patients in the FH and non-FH groups were matched using the propensity score matching (PSM) method to adjust for differences in baseline variables. The propensity score for an individual was calculated given the covariates of age, gender, smoking, alcohol consumption, dysphagia, radicality of surgery, adjuvant therapy, pathological differentiation, tumor location, and pathological stage using a multivariable logistic regression model. A computerized technique was used for the nearest available score matching with the caliper value of 0.0001 and no replacement selection, and 215 patients in the non-FH group were individually matched to patients in the FH group.

### Statistical analysis

Statistical analysis was performed using SPSS 16.0 for the Windows software system (SPSS Inc., Chicago, IL, USA). Chi-square tests were performed to evaluate the associations between clinicopathological variables and FH. Survival curves were calculated using the Kaplan–Meier method and compared using log-rank tests. Multivariate analysis was performed using Cox’s proportional hazards regression model with a forward stepwise procedure (the entry and removal probabilities were 0.05 and 0.10, respectively). We tested the proportional hazards assumption by the Schoenfeld residuals test to determine if the test was not statistically significant for each of the covariates, as well as the global test. Therefore, we assumed proportional hazards. A difference was considered significant if *p* < 0.05 (two-tailed).

## Results

### Characteristics of patients by family history of cancer before and after matching

Before matching, a total of 1,322 consecutive patients with ESCC were included in the study. Of these patients, 280 (21.2%) had FH, and 1,042 (78.8%) did not have FH. Among patients with FH, 183 (13.8%) had FH-EC, and 97 (7.4%) had FH-NEC. Further, 238 (18.0%) patients had FH-DC, and 42 (3.2%) had FH-NDC. Moreover, 260 (19.7%) patients had FH-FD, and 20 (1.5%) had a second- or third-degree family history of any cancer (FH-NFD); 235 (17.8%) patients had only one relative with a history of any cancer, and 45 (3.4%) had more than one relative with a history of any cancer. The baseline characteristics of patients were compared between the FH and non-FH groups (**Table 1**). Compared to the non-FH group, the FH group was associated with no dysphagia symptom (53.6% vs. 41.3%, *p* < 0.001), early pathological T stage (41.8% vs. 31.3%, *p* = 0.001), LN-negative status (54.6% vs. 46.8%, *p* = 0.022), and early pathological stage (62.5% vs. 53.2%, *p* = 0.006). However, no significant difference was noted in age, gender, smoking status, alcohol status, radicality of surgery, differentiation, tumor location, or receiving adjuvant therapy between the groups.

**Table 1:** The clinical and pathologic characteristics at baseline.

Characteristic	No. of patients (%) before PSM				No. of patients (%) after PSM		
	Overall (n = 1322)	Non FH (n = 1042)	FH (n = 280)	P-Value	Non FH (n = 215)	FH (n = 215)	P-Value
**Age**				0.148			1.000
≤58 years	803 (60.7)	622 (59.7)	181 (64.6)		139 (64.7)	140 (65.1)	
>58 years	519 (39.3)	420 (40.3)	99 (35.4)		76 (35.3)	75 (34.9)	
**Gender**				0.628			0.910
Females	296 (22.4)	230 (22.1)	66 (23.6)		52 (24.2)	50 (23.3)	
Males	1026 (77.6)	812 (77.9)	214 (76.4)		163 (75.8)	165 (76.7)	
**Smoking**				0.575			0.919
Never	472 (35.7)	368 (35.3)	104 (37.1)		73 (33.9)	75 (34.9)	
Ever (former + current)	850 (64.3)	674 (64.7)	176 (62.9)		142 (66.1)	140 (65.1)	
**Alcohol**				0.103			1.00
Never	868 (65.7)	696 (66.8)	172 (61.4)		137 (63.7)	138 (64.2)	
Ever (former + current)	453 (34.3)	346 (33.2)	108 (38.6)		78 (36.3)	77 (35.8)	
**Dysphagia**				**<0.001**			0.772
No	580 (43.9)	430 (41.3)	150 (53.6)		105 (48.8)	109 (50.7)	
Yes	742 (56.1)	612 (58.7)	130 (46.4)		110 (51.2)	106 (49.3)	
**Radicality of surgery**				0.509			1.000
R0	1265 (95.7)	999 (95.9)	266 (95.0)		212 (98.6)	213 (99.1)	
R1	57 (4.3)	43 (4.1)	14 (5.0)		3 (1.4)	2 (0.9)	
**pT stage**				**0.001**			0.921
T1-2	433 (33.5)	326 (31.3)	117 (41.8)		85 (39.5)	83 (38.6)	
T3-4	879 (66.5)	716 (68.7)	163 (58.2)		130 (60.5)	132 (61.4)	
**pN stage**				**0.022**			0.847
N0	641(48.5)	488 (46.8)	153 (54.6)		117 (54.4)	114 (53.0)	
N1-3	681(51.5)	554 (53.2)	127 (45.4)		98 (45.6)	101 (47.0)	
**Differentiation**				0.334			0.914
G1-2	942 (71.3)	749 (71.9)	193 (68.9)		156 (72.6)	158 (73.5)	
G3	380 (28.7)	293 (28.1)	87 (31.1)		59 (27.4)	57 (26.5)	
**Tumor location**				0.629			0.906
Upper	295 (22.3)	227 (21.8)	68 (24.3)		43 (20.0)	44 (20.5)	
Middle	907 (68.6)	721 (69.2)	186 (66.4)		162 (75.4)	159 (74.0)	
Lower	120 (9.1)	94 (9.0)	26 (9.3)		10 (4.6)	12 (5.5)	
**TNM stage**				**0.006**			0.843
I-II	729 (55.1)	554 (53.2)	175 (62.5)		133 (61.9)	130 (60.5)	
III	593 (44.9)	488(46.8)	105(37.5)		82 (38.1)	85 (39.5)	
**Adjuvant therapy**				0.414			1.000
No	1035 (78.3)	821 (78.8)	214 (76.4)		178 (82.8)	177 (82.3)	
Yes	287 (21.7)	221 (21.2)	66 (23.6)		37 (17.2)	38 (17.7)	

PSM, Propensity Score Matching; FH, family history of cancer.

Bold values are statistically significant (P < 0.05).

After matching, 215 in the FH group and 215 patients in the non-FH group were included, and the baseline characteristics were well-balanced between the two groups (**Table 1**).

### Prognostic value of family history of cancer in esophageal squamous cell carcinoma after propensity score matching

**Figure 1:**
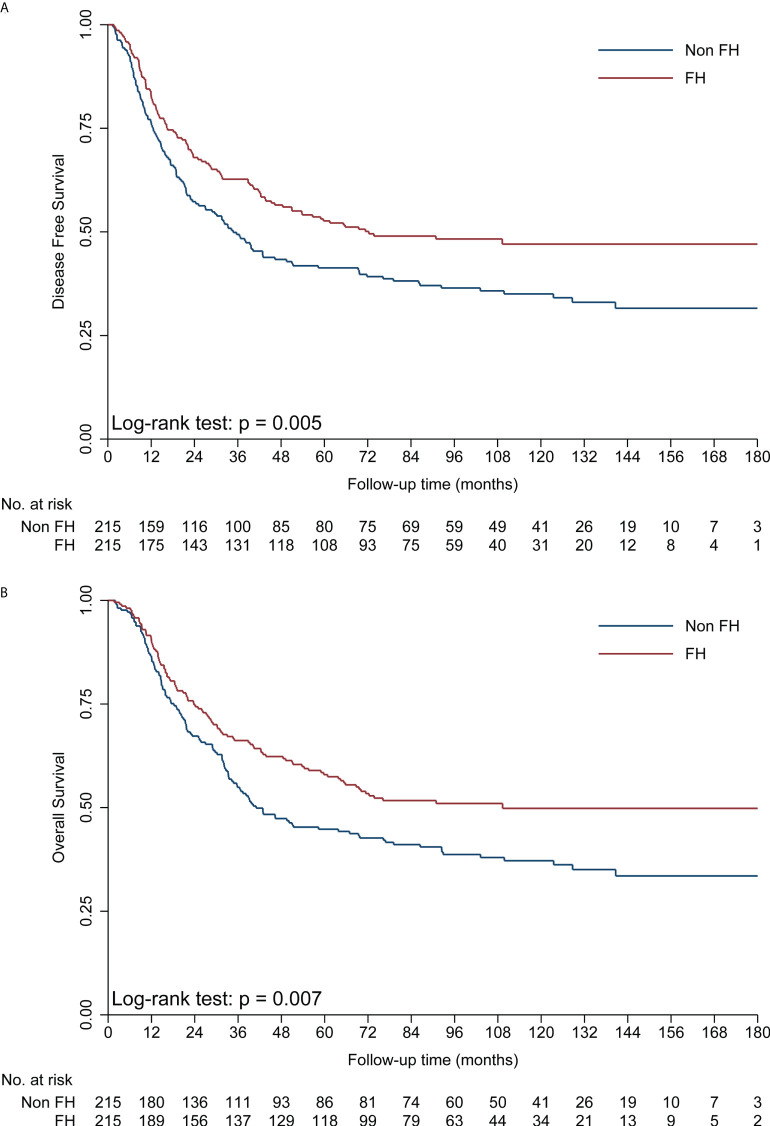
Kaplan–Meier curves showing a significant difference in DFS **(A)** and OS **(B)** in the FH and non-FH groups. DFS, disease-free survival; FH, family history of cancer; OS, overall survival.

The median time of follow-up was 107.1 months. Up to the last day of follow-up, 102 of 215 (47.4%) patients with FH died, and 129 of 215 (60.0%) patients without FH died. Univariate survival analysis showed that patients with FH had a significantly better DFS (hazard ratio [HR] = 0.70, 95% CI: 0.54–0.90, *p* = 0.005, **Table 2**; **Figure 1**) and OS (HR = 0.70, 95% CI: 0.54–0.91, *p* = 0.007, **Table 2**; **Figure 1**) than those without FH. As shown in **Table 2**, being male, a history of smoking, alcohol consumption, absence of radical resection, cancer in the upper thoracic esophagus, advanced pathological T stage, LN metastasis, and poorly differentiated cancer (G3) were associated with significantly shorter DFS and OS rates (*p* < 0.05). However, no significant association between age or adjuvant therapy with DFS or OS was observed in patients with ESCC.

**Table 2:** Univariate and multivariate analysis for DFS, OS in patients with ESCC after PSM.

Prognostic factor	Univariate survival analysis				Multivariate survival analysis			
		DFS		OS		DFS		OS
	HR (95%CI)	P-Value	HR (95%CI)	P-Value	HR (95%CI)	P-Value	HR (95%CI)	P-Value
**Age**								
** ≤58 years (n=279)**	1		1					
** >58 years (n=279)**	1.12 (0.86,1.45)	0.394	1.21 (0.93, 1.57)	0.167				
**Gender**								
** Male (n=328)**	1		1		1		1	
** Female (n=102)**	0.57 (0.41,0.80)	**0.001**	0.60 (0.43, 0.83)	**0.002**	0.76 (0.46, 1.26)	0.283	0.93 (0.54, 1.60)	0.797
**Smoking**								
** Never (n=148)**	1		1		1		1	
** Ever (n=282)**	1.46 (1.11, 1.92)	**0.008**	1.53 (1.15, 2.04)	**0.003**	1.22 (0.77, 1.93)	0.395	1.45 (0.88, 2.38)	0.145
**Alcohol**								
** Never (n=275)**	1		1		1		1	
** Ever (n=155)**	1.35 (1.05, 1.75)	**0.021**	1.43 (1.10, 1.86)	**0.008**	1.09 (0.81, 1.48)	0.565	1.17 (0.86, 1.61)	0.314
**Radicality of surgery**								
** R1 (n=5)**	1		1		1		1	
** R0 (n=425)**	0.33 (0.12, 0.88)	**0.027**	0.27 (0.10-0.73)	**0.009**	0.42 (0.15, 1.21)	0.108	0.32 (0.11,0.94)	**0.038**
**pT stage**								
** T1-2 (n=168)**	1		1		1		1	
** T3-4 (n=262)**	1.66 (1.27, 2.17)	**<0.001**	1.74 (1.32 2.29)	**<0.001**	1.39 (1.05, 1.83)	**0.022**	1.51 (1.13, 2.01)	**0.005**
**pN stage**								
** N0 (n=231)**	1		1		1		1	
** N1-3 (n=199)**	2.09 (1.62, 2.69)	**<0.001**	2.06 (1.58, 2.67)	**<0.001**	2.04 (1.52, 2.73)	**<0.001**	2.04 (1.51, 2.75)	**<0.001**
**Differentiation**								
** G1-2 (n=314)**	1		1		1		1	
** G3 (n=116)**	1.51 (1.14, 1.98)	**0.003**	1.50 (1.13, 1.98)	**0.005**	1.38 (1.05,1.83)	**0.023**	1.38 (1.03,1.84)	**0.029**
**Tumor location**								
** Upper (n=87)**	1		1		1		1	
** Middle (n=321)**	0.83 (0.62, 1.12)	0.221	0.78 (0.58, 1.06)	0.116	0.77 (0.57, 1.05)	0.105	0.74 (0.54, 1.01)	0.060
** Lower (n=22)**	0.47 (0.23, 0.94)	**0.033**	0.41 (0.20, 0.87)	**0.020**	0.41 (0.20 0.84)	0.015	0.37 (0.17, 0.79)	0.010
**Adjuvant therapy**	1		1		1		1	
** No (n=355)**								
** Yes (n=75)**	1.37 (0.99, 1.89)	0.054	1.24 (0.88, 1.73)	0.216	0.81 (0.57, 1.15)	0.241	0.72 (0.50, 1.04)	0.082
**FH**								
** Non (n=215)**	1		1		1		1	
** FH (n=215)**	0.70 (0.54, 0.90)	**0.005**	0.70 (0.54, 0.91)	**0.007**	0.65 (0.51 0.84)	**0.001**	0.66 (0.51, 0.86)	**0.002**

Age, continuous variable; Gender, categorical variable; Smoking, categorical variable; Alcohol, categorical variable; Radicality of surgery, categorical variable; pT stage, categorical variable; pN stage, categorical variable; Differentiation, categorical variable; Tumor location, categorical variable; Adjuvant therapy, categorical variable; FH, categorical variable.

PSM, Propensity Score Matching; FH, family history of cancer.

Bold values are statistically significant (P < 0.05).

The Cox’s proportional hazards regression suggested that FH was an independent favorable prognostic factor in operable EC (**Table 2**). In the final multivariate survival analysis with adjustment for prognostic factors, patients with FH had 35% lower risks of disease progression (HR = 0.65, 95% CI: 0.51–0.84, *p* = 0.001) and 34% lower risk of death (HR = 0.66, 95% CI: 0.51–0.86, *p* = 0.002) than those without FH.

**Figure 2:**
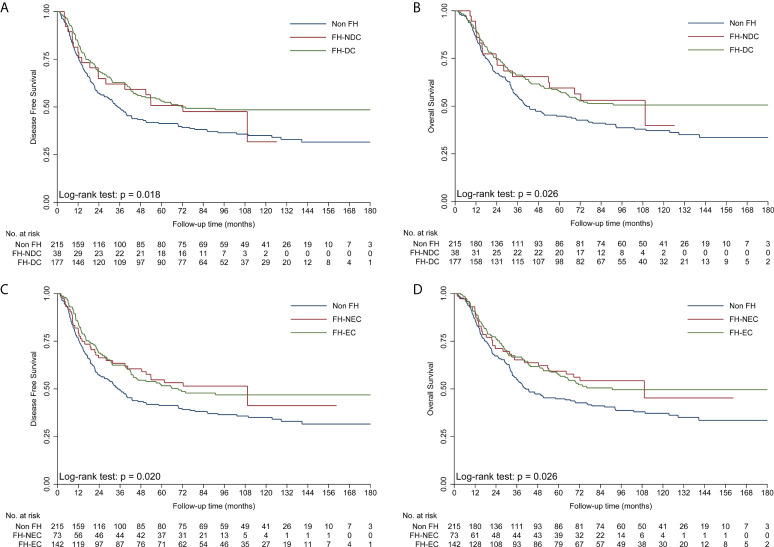
Kaplan–Meier curves showing better DFS **(A)** and OS **(B)** in the FH-DC group than in the non-FH group, but not in the FH-NDC group. Kaplan–Meier curves showing better DFS **(C)** and OS **(D)** in the FH-EC and non-FH groups. DFS, disease-free survival; FH, family history of cancer; FH-DC, family history of digestive tract cancer; FH-EC, family history of esophageal cancer; FH-NDC; family history of non-digestive tract cancer; FH-NFD, family history in non-first-degree; OS, overall survival.

**Figure 3:**
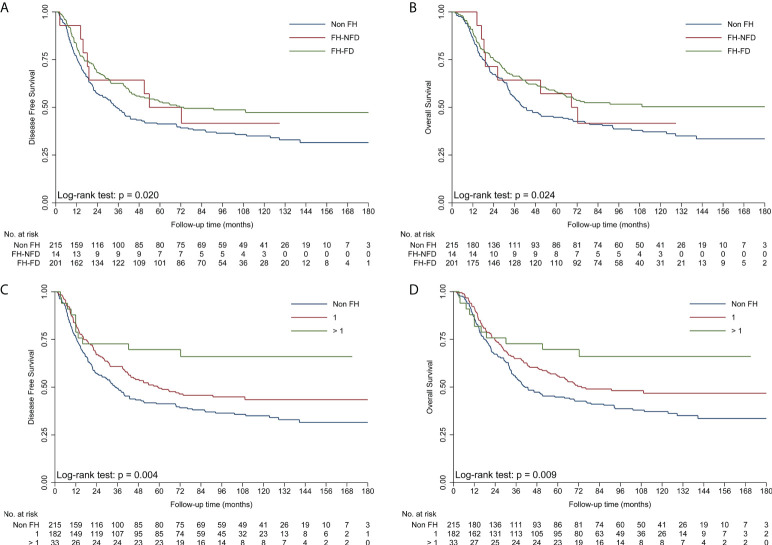
Kaplan–Meier curves showing better DFS **(A)** and OS **(B)** in the FH-FD group than in the non-FH group, but not in the FH-NFD group; Kaplan–Meier curves showing better DFS **(C)** and OS **(D)** in patients with more than one relative affected by cancer than in patients in the non-FH group. DFS, disease-free survival; FH, family history of cancer; FH-DC, family history of digestive tract cancer; FH-NDC; family history of non-digestive tract cancer; FH-NFD, family history in non-first-degree; non-FH, no family history of cancer; OS, overall survival.

To obtain insights into the influence of FH on survival, further analyses were performed for different types of FH in ESCC patients (**Table 3**). First, patients with FH-DC had better DFS and OS than those without FH, while patients with FH-NDC had the same survival as those without FH in the univariate and multivariate survival analyses (**Table 3**; **Figures 2A, B**). Second, compared to patients in the non-FH group, those in the FH-EC group were associated with improved DFS and OS in univariate and multivariate survival analyses, but FH-NEC was only associated with improved DFS and OS after adjustment of prognostic factors in the multivariate survival analysis (**Table 3**; **Figures 2C, D**). Third, patients with FH-FD were associated with improved DFS and OS rates compared to those without FH in the univariate and multivariate survival analyses, but patients with FH-NFD did not show this association (**Table 3**; **Figures 3A, B**). Fourth, patients with more than one relative affected by cancer had significantly favorable DFS and OS rates as compared to those without FH (**Table 3**; **Figures 3C, D**). However, for patients with only one relative affected by cancer, improvement in OS and DFS rates was observed in the multivariate survival analysis but not for improvement in DFS in the univariate analysis. Therefore, a trend for reduction of overall mortality and disease progression was observed with an increase in the number of affected family members.

**Table 3:** Univariate and multivariate survival analysis for different types of family history of cancer in ESCC after PSM.

Prognostic factor	Univariate survival analysis				Multivariate survival analysis*			
	DFS		OS		DFS		OS	
	HR (95%CI)	P-Value	HR (95%CI)	P-Value	HR (95%CI)	P-Value	HR (95%CI)	P-Value
**FH-DC**								
on FH (n=215)	1		1		1		1	
FH-NDC (n=38)	0.77 (0.48, 1.24)	0.284	0.71 (0.43, 1.18)	0.190	0.71 (0.44, 1.14)	0.151	0.67 (0.40, 1.12)	0.125
FH-DC (n=177)	0.68 (0.52, 0.89)	**0.005**	0.70 (0.53, 0.92)	**0.010**	0.64 (0.49, 0.84)	**0.001**	0.66 (0.50, 0.87)	**0.003**
**FH-EC**								
Non FH (n=215)	1		1		1		1	
FH-NEC (n=73)	0.69 (0.48, 1.00)	0.053	0.69 (0.47,1.01)	0.059	0.59 (0.40,0.86)	**0.006**	0.59 (0.40,0.87)	**0.008**
FH-EC (n=142)	0.70(0.53, 0.93)	**0.014**	0.70 (0.53,0.94)	**0.019**	0.69 (0.51,0.92)	**0.011**	0.70 (0.52,0.94)	**0.019**
FH-FD								
Non FH (n=215)	1		1		1		1	
FH-NFD (n=14)	0.75 (0.37, 1.54)	0.439	0.81 (0.40, 1.66)	0.566	0.79 (0.39, 1.63)	0.525	0.82 (0.40,1.69)	0.596
FH-FD (n=201)	0.69 (0.54, 0.90)	**0.006**	0.69 (0.53,0.90)	**0.007**	0.64 (0.49,0.84)	**0.001**	0.65 (0.50,0.85)	**0.002**
Number of Relative								
Non FH (n=215)	1		1		1		1	
1 (n=182)	0.76 (0.58,0.98)	0.036	0.75 (0.57,0.98)	**0.035**	0.69 (0.53,0.89)	**0.005**	0.69 (0.52,0.90)	**0.007**
>1 (n=33)	0.42 (0.22,0.77)	**0.005**	0.45 (0.24,0.84)	**0.012**	0.46 (0.25,0.85)	**0.014**	0.51 (0.28,0.95)	**0.034**

PSM, Propensity Score Matching; FH, family history of cancer; FH-DC, family history of digestive tract cancer; FH-NDC, family history of non digestive tract cancer; FH-EC, family history of esophageal cancer; FH-NEC, family history of non esophageal cancer; FH-FD, family history of cancer in first-degree; FH-NFD, family history of cancer in non first-degree.

* Multivariate survival analysis were adjusted by gender, smoking, alcohol, radicality of surgery, pT stage, pN stage, differentiation, tumor location and adjuvant therapy.

Bold values are statistically significant (P < 0.05).

## Discussion

In this study, before PSM, we found that FH was more likely to be associated with the early pathological stage, including T and N stages, compared to the absence of FH. After PSM, patients with FH, especially with FH-DC, FH-EC, FH-FD, and more than one relative affected by cancer, had a significant reduction in the risk of death and recurrence after adjustments for known prognostic factors.

Numerous studies have shown that a family history of EC increases the risk of developing this disease (8–11). However, the effect of FH on the prognosis of patients with operable ESCC has been rarely studied and is unclear. In our current study, patients in the FH and non-FH groups were matched using PSM to adjust for differences in baseline variables, and FH was found to be a favorable independent prognostic factor for ESCC. Our findings were quite similar to those of previous studies in patients with breast (4), colorectal (5), and gastric cancers (6), as well as in those with nasopharyngeal carcinoma (7). However, our results were inconsistent with those of previous studies on EC (12–14). One study with 1,715 patients from Northern China reported that a family history of upper gastrointestinal cancer was significantly associated with poor survival rate after surgery in early-stage patients above the age of 50 years but was not significantly associated with all ESCC patients (12). Another study with 1,553 patients from Western China reported that a family history of EC negatively affected survival among ESCC patients who had undergone surgery (13). Another study from Southern China showed that 479 ESCC patients with first-degree FH, especially digestive tract cancer, had unfavorable survival after radiotherapy (14). These inconsistencies may be because of the variations in the populations enrolled, treatments administered, definitions of family history, and sample sizes. Moreover, FH was significantly associated with an early stage in these three previous studies. However, in these studies, the baseline characteristics were not well balanced between groups to greatly influence the prognostic value of FH. In our study, we also found a similar association but used PSM to reduce bias. After PSM, we showed that FH was a favorable independent prognostic factor in patients with ESCC. We then further examined the effect of FH-EC, FH-DC, and FH-FD on survival in patients with ESCC. Consequently, significant improvement in DFS and OS was noted in patients with FH-EC, FH-DC, and FH-FD, whereas the effect of family history was not observed for patients with FH-NDC and FH-NFD when compared to those without FH. Additionally, we also examined the effect according to the number of affected relatives. A significant trend for reduction in risk of death and recurrence was observed with an increase in the numbers of affected family members, which is consistent with results in previous studies in breast (4), colorectal (5), and gastric cancers (6), as well as nasopharyngeal carcinoma (7). To the best of our knowledge, our study is the first large cohort study to systematically elucidate the prognostic value of FH in ESCC patients.

The mechanisms underlying the association between FH and the survival of patients with ESCC are still unknown. The prognostic advantage of FH might be attributed to its significant association with early-stage cancer not only in our study but also in other studies (6, 7, 12–14). We also found that patients with FH were less likely to have dysphagia that indicated advanced-stage disease. These findings suggest that individuals with FH may be more likely to undergo regular cancer surveillance, thereby being diagnosed at an early stage of cancer. Previous studies have already shown that patients with FH were more likely to undergo prostate or cervical cancer screening (17, 18). No significant difference between FH and adjuvant therapy was noted in our study, which indicated that adjuvant therapy had no effect on the prognostic advantage for patients with FH. We believe that the bias from the screening effect could be minimized by using PSM analysis and adjustment for pathological T stage, LN metastasis, and adjuvant therapy in the multivariate survival analysis.

Health-related behavior such as smoking status and alcohol consumption might be also responsible for the survival difference, as reported by Han et al. (6) in patients with gastric cancer. Smoking and alcohol status were associated with poor survival of EC patients in our current study and previous studies (16, 19). Patients with FH were more likely to make positive behavioral changes, such as quitting smoking, and positive dietary changes (20, 21). However, FH and smoking or alcohol status were significantly associated with our study. Additionally, smoking and alcohol status were adjusted in the multivariate survival analysis to minimize their effects on the prognostic value of FH.

Genetic predisposition has proven to play an important role in the younger age of onset and patients with multiple primary ESCC with FH (22). A previous study showed that *RHBDF2* mutations are closely associated with tylosis, a familial EC syndrome (23). Another study found that *BRCA2* mutation is more frequent in ESCC patients with FH than in those without FH, suggesting that *BRCA2* may play a role in genetic susceptibility to familial ESCC (24). We inferred that genetic factors might play an intrinsic role in the prognostic value of FH. In our current study, patients with FH-FD and FH-DC rather than patients with FH-NFD or FH-NDC had better survival. Moreover, we noted a significant trend for reduction in risk of death and recurrence with increasing numbers of affected family members. These findings indicate that patients with FH-FD, FH-DC, and more than one relative affected by cancer were more likely genetically susceptible than those without FH. Thus, further basic research is needed to fully elucidate the potential mechanisms of the impact of FH on prognosis.

We acknowledge several limitations of this study. First, our study was a single-institution retrospective study, which may have led to selection bias. However, we used PSM analysis to adjust for differences in baseline variables between the two groups of patients. The results remained unchanged after adjustment for known prognostic factors. Second, FH data were based on self-reports and may lead to the misclassification of family history status, especially under-reporting the second-degree family history. However, a previous study has proven such data to be reliable (25). Third, selection bias may have been introduced because patients with metastatic disease and those with unresectable ESCC were excluded. Fourth, the data on socioeconomic status and education degree were not collected to explore their effect on the prognostic advantage for patients with FH in this study.

## Conclusion

FH is a favorable independent prognostic factor for patients with ESCC after esophagectomy. Patients with FC, especially those with FH-DC, FH-EC, FH-FD, and more than one relative affected by cancer, had better survival than those without FH. Further prospective studies of large cohorts of patients are necessary to confirm these results.

## Data availability statement

The raw data supporting the conclusions of this article will be made available by the authors, without undue reservation.

## Author contributions

SZ participated in drafting the manuscript. SZ, JC and XC participated in writing the manuscript. KW participated in the acquisition of data. BL participated in the data analysis. QL, ZT and YZ designed the study and revised the manuscript. All authors contributed to the article and approved the submitted version.

## Funding

This study was supported by grants from the National Science Foundation of China (Grant No. 82103077 to SZ) and the Natural Science Foundation of Guangdong Province, China (2021A1515011775 to SZ).

## Acknowledgments

We would like to thank the patients and family members who gave their consent to present their data in this study.

## Conflict of interest

The authors declare that the research was conducted in the absence of any commercial or financial relationships that could be construed as a potential conflict of interest.

## Publisher’s note

All claims expressed in this article are solely those of the authors and do not necessarily represent those of their affiliated organizations, or those of the publisher, the editors and the reviewers. Any product that may be evaluated in this article, or claim that may be made by its manufacturer, is not guaranteed or endorsed by the publisher.
